# Interim Report by King's Fund Policy Committee

**Published:** 1921-04-23

**Authors:** 


### THE PRESERVATION OF THE VOLUNTARY SYSTEM.

### Interim Report by King's Fund Policy Committee.

After the issue of the report of King Edward's Fund on January 26, containing the thirteen resolutions dealing with the policy to be recommended for the preservation of the voluntary system of hospital management and control, a Committee was appointed to work out the details of the policy and to take steps to recommend it to the Government, to the Committee of Inquiry presided over by Lord Cave, to the hospitals, and to the public. This Committee has now made an interim report, dated April 4, and signed by Lord Stuart of Wortley, the chairman. The principal features are as follows:—

To the end of 1920 the difficulties of the hospitals were not due to any falling off in receipts. On the contrary, the income from normal sources at 113 London hospitals increased from £1,479,000 in 1913 to £2,117,000 in 1919 and £2,447,000 in 1920. Unfortunately, the expenditure increased still more, rising from £1,189,000 in 1913 to £2,348,000 in 1919 and £2,841,000 in 1920. The result was an aggregate net deficit of about £394,000 on the working of the year 1920, and accumulated bank loans of £543,000 at the end of the year.

The grants from the King's Fund Emergency Distribution and the National Relief Fund almost exactly wiped out this deficit; but these sources of income are non-recurrent. They are not included in the above figures of income, though they have, of course, been taken into account in arriving at the total of bank loans.

### Alternative Remedies.

The supplementary income must be adequate to meet the present deficits at hospitals that have deficits, without piling up surpluses at some hospitals and leaving others in difficulties; it must be capable of expanding to meet the cost of necessary extensions and developments, and it must not endanger voluntary management and control or the interests of the sick poor. It may be argued that substantially the choice is limited to two alternatives:—

(*a*) Payment in some form or other for specific work (which might include payment by means of voluntary or compulsory insurance), and

(*b*) Assistance from public funds proportioned in some way to voluntary contributions.

Payment for specific work includes payment by public authorities for particular classes of patients—e.g., tuberculosis, venereal, war pensioners, maternity, school children, etc.—and payments by actual or prospective patients.

Receipts from public authorities increased from £11,000 in 1913 to £190,000 (apart from naval and military patients) in 1919 and £311,000 in 1920.

Payments made by or for patients have increased from £78,000 in 1913 to £124,000 in 1919 and £230,000 in 1920. Larger sums are expected in 1921, as many hospitals did not develop either system till late in 1920.

It is obvious that many patients who cannot pay during illness could, nevertheless, afford to make provision for hospital treatment by some form of voluntary insurance. A comprehensive scheme has begun to be put into operation in Sussex. The King's Fund Policy Committee is not at present prepared to endorse this scheme, especially as it has not yet been considered by the Hospital Saturday Fund.

### National Insurance Funds.

With regard to National Insurance, Lord Cave's Committee, in its interim report of March 9, suggests that the Approved Societies should recognise the services of the hospitals by devoting a portion of their disposable surpluses to their assistance; and it is possible that a considerable part, or the whole, of the present hospital deficit might be wiped out in this way, and that the expenses of the maintenance of insured patients in the hospitals in the future might be met by definite provision for hospital benefit being made in advance out of the contributions of the employers, the employed, and the State. The King's Fund Policy Committee considers that both forms of insurance should be explored, in order to see whether the problem could be solved if the voluntary subscriptions of the charitable public were supplemented by a graduated system of patients' payments from those able to pay at the time of illness and by insurance on the part of those who are unable so to do; the hospitals agreeing to limit free treatment, except in emergencies, to those unable to pay or to insure.

The Policy Committee has not yet given consideration to the alternative of State or municipal assistance to encourage voluntary contributions, but the possibilities of this alternative should be investigated.

### The Question of Emergency Assistance.

Last year the financial difficulty became acute in May or June, and, had it not been for the emergency distribution of £250,000 by the King's Fund, some important hospitals might have been obliged either to close down or to come on public funds. As the deficit in 1920 was much larger than in 1919, and in view of existing circumstances, a similar and more severe crisis may occur this year before there has been time to work out a permanent remedy.

If emergency assistance again becomes necessary, any further depletion of the reserves of the King's Fund is most strongly deprecated, except possibly as an advance with a guarantee that the money would be replaced. The special efforts made last year reduced the available reserves by about one-fourth and the permanent income by about £16,000, and any repetition would seriously weaken the central fund.

Some other method would have to be found. One alternative would be Government assistance in some form which would be wholly outside the sphere of any scheme of permanent remedies, and which, therefore, would not prejudge any of the questions already discussed in this report. It is possible that the recommendation already made by Lord Cave's Committee on the subject of the surpluses now in the hands of the Approved Societies may have the result of producing sufficient money to avoid any immediate collapse.

### Central Body.

The document of January 26, on which the Council invited the observations of the hospitals and the British Hospitals Association, contained resolutions as to the necessity for some central organisation and as to the suitability of the King's Fund to become a central administrative body for the Metropolitan area.

It is understood that in using the term "administrative body" the Council mean that the powers and functions of the central body should go beyond those of a mere distributing body on the one hand, or a consultative body on the other; and that it is better for the voluntary system that such a central body should be the King's Fund than that it should take any of the other possible forms that have as yet been contemplated. For the King's Fund, while interested in the voluntary system, for the maintenance of which it exists, would be independent both of the hospitals and of agencies outside the voluntary system.

### Increase of Hospital Accommodation.

The amounts already received and promised for all schemes of extension, besides £310,000 in grants from the distribution of surplus Red Cross funds and from the ordinary distribution of the King's Fund, is about £500,000.

It is evident that the financial problem of making provision for even the most urgent development of hospital accommodation is a serious one; and the possibility of saving capital expenditure by making the best use of any existing buildings requires the fullest consideration.

